# Whole body and hematopoietic ADAM8 deficiency does not influence advanced atherosclerotic lesion development, despite its association with human plaque progression

**DOI:** 10.1038/s41598-017-10549-x

**Published:** 2017-09-15

**Authors:** Kosta Theodorou, Emiel P. C. van der Vorst, Marion J. Gijbels, Ine M. J. Wolfs, Mike Jeurissen, Thomas L. Theelen, Judith C. Sluimer, Erwin Wijnands, Jack P. Cleutjens, Yu Li, Yvonne Jansen, Christian Weber, Andreas Ludwig, Jacob F. Bentzon, Jörg W. Bartsch, Erik A. L. Biessen, Marjo M. P. C. Donners

**Affiliations:** 1 Department of Pathology, CARIM, Maastricht University, Maastricht, The Netherlands; 2 Institute for Cardiovascular Prevention, Ludwig-Maximilians-University Munich, Munich, Germany; 3 Department of Molecular Genetics, CARIM, Maastricht University, Maastricht, The Netherlands; 4 Department of Medical Biochemistry, AMC, Amsterdam, Netherlands; 5 Department of Biochemistry, CARIM, Maastricht University, Maastricht, Netherlands; 6 DZHK (German Centre for Cardiovascular Research), partner site Munich Heart Alliance, Munich, Germany; 7 Institute of Pharmacology and Toxicology, RWTH Aachen University, Aachen, Germany; 8 Centro Nacional de Investigaciones Cardiovasculares Carlos III, Madrid, Spain; 9 Department of Clinical Medicine, Aarhus University, Aarhus, Denmark; 10 Department of Neurosurgery, Philipps University Marburg, Marburg, Germany; 11 Institute for Molecular Cardiovascular Research, RWTH Aachen, Aachen, Germany

## Abstract

Although A Disintegrin And Metalloproteinase 8 (ADAM8) is not crucial for tissue development and homeostasis, it has been implicated in various inflammatory diseases by regulating processes like immune cell recruitment and activation. ADAM8 expression has been associated with human atherosclerosis development and myocardial infarction, however a causal role of ADAM8 in atherosclerosis has not been investigated thus far. In this study, we examined the expression of ADAM8 in early and progressed human atherosclerotic lesions, in which ADAM8 was significantly upregulated in vulnerable lesions. In addition, ADAM8 expression was most prominent in the shoulder region of human atherosclerotic lesions, characterized by the abundance of foam cells. In mice, *Adam8* was highly expressed in circulating neutrophils and in macrophages. Moreover, ADAM8 deficient mouse macrophages displayed reduced secretion of inflammatory mediators. Remarkably, however, neither hematopoietic nor whole-body ADAM8 deficiency in mice affected atherosclerotic lesion size. Additionally, except for an increase in granulocyte content in plaques of ADAM8 deficient mice, lesion morphology was unaffected. Taken together, whole body and hematopoietic ADAM8 does not contribute to advanced atherosclerotic plaque development, at least in female mice, although its expression might still be valuable as a diagnostic/prognostic biomarker to distinguish between stable and unstable lesions.

## Introduction

Atherosclerosis is a lipid-driven chronic inflammatory disease, initiated by endothelial dysfunction, resulting in the subendothelial accumulation and modification of circulating lipoprotein particles, together with the recruitment of leukocytes within the vessel wall. These modified lipoproteins are internalized by macrophages, forming foam cells which eventually become apoptotic. Progressed atherosclerotic lesions are characterized by the formation of a fibrous cap and a lipid rich necrotic core^1^. These lesions may, upon rupture, lead to local thrombosis, the major cause of clinical events such as myocardial infarction and stroke^2^.

A Disintegrin And Metalloproteinases (ADAMs) are a family of transmembrane proteases which play a role in modulating inflammatory responses^3^. Their role in cardiovascular diseases (CVD)/ atherosclerosis is emerging as evidenced by several recent publications showing that ADAM10 modulates atherosclerotic plaque composition^4^, while ADAM15 contributes to lesion development^5^ and ADAM17 provides with atherosclerosis resistance^6, 7^. Among several members of this family, ADAM8 exhibits sheddase activity, enabling cleavage of atherosclerosis related cell surface proteins, including the inflammatory molecules L-selectin, P-selectin glycoprotein ligand-1 (PSGL-1), tumor necrosis factor (TNF), TNF receptor 1 and vascular cell adhesion molecule 1^8, 9^. ADAM8 is highly expressed in most cells of hematopoietic origin, and in the brain, bone, lung and thymus^10–15^. Despite being widely expressed, mice deficient in ADAM8 have a normal development with no overt phenotype^15^. With respect to pathologies, ADAM8 levels increase in cancer and inflammatory diseases of the lung, central nervous system, bone and joints, and its expression positively correlates with disease severity^11, 16–18^.

ADAM8 expression was also upregulated in human atherosclerotic plaques compared to non-atherosclerotic control vessels^19^. Moreover, this study showed specific ADAM8 polymorphisms (rs2995300C and rs2275725A) to be associated with atherosclerosis development and myocardial infarction in two independent human cohorts. However, it is unclear whether ADAM8 is also causally involved in atherosclerosis development. In this study, we investigated whether ADAM8 plays a role in the development and progression of atherosclerotic lesions. Although we show that ADAM8 expression is associated with lesion progression in human disease, genetic ablation of ADAM8 both in the hematopoietic compartment as well as at whole-body level did not affect advanced atherosclerosis development in female mice.

## Results

### ADAM8 expression increases with atherosclerotic plaque progression in humans and is mainly associated with foam cell-rich regions

**Figure 1:**
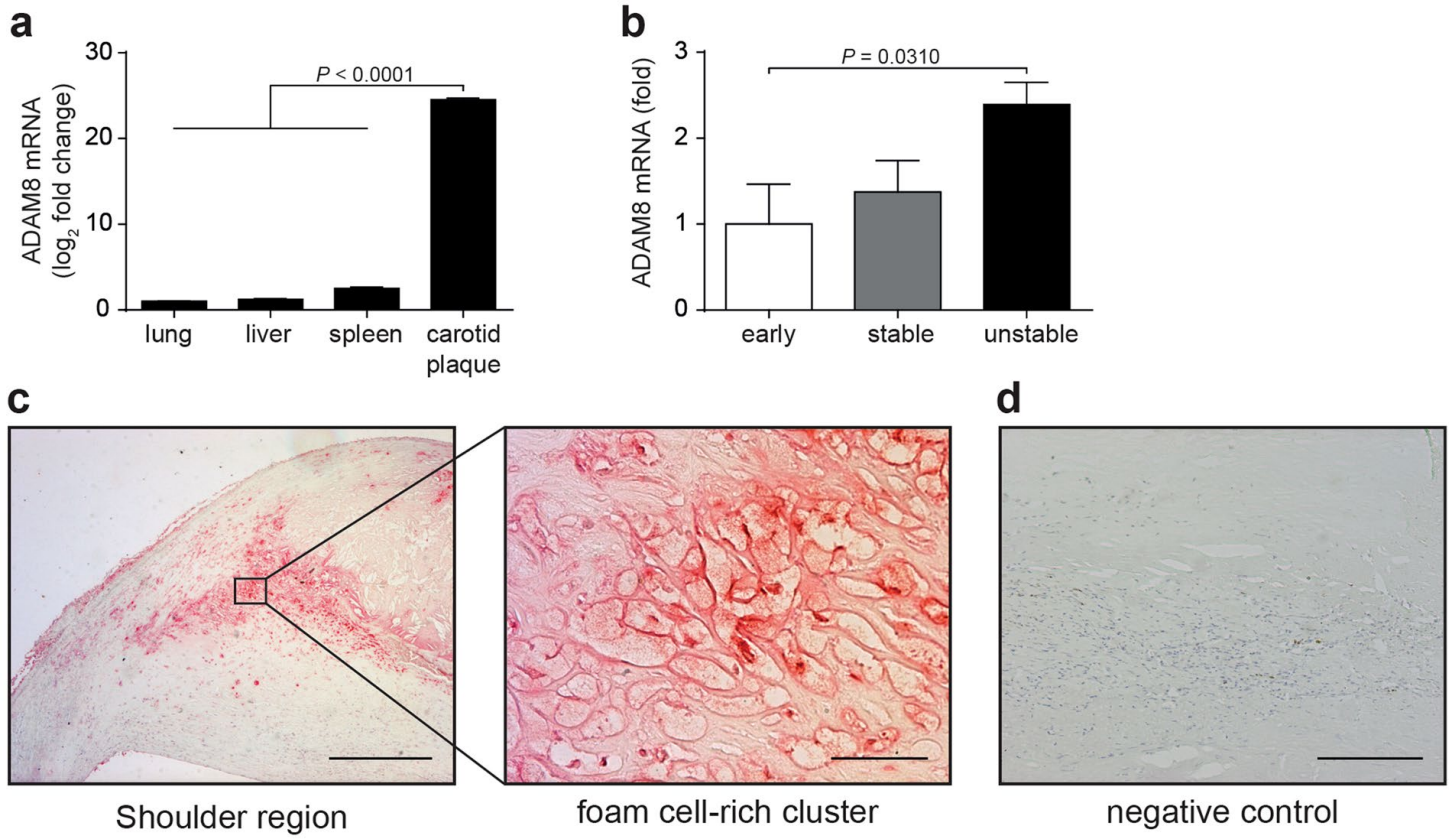
ADAM8 expression in human atherosclerotic lesions. (**a**) Microarray expression profiling of carotid atherosclerotic plaque macrophages and lung, liver and spleen macrophages (*n* = 4 patients per tissue, nonparametric Mann-Whitney *U* test). (**b**) Relative expression of ADAM8 mRNA in early, advanced stable and unstable human plaques. Values have been corrected for GAPDH expression (*n* = 5–6) and ADAM8 expression levels in early plaques were normalized to 1 (one way analysis of variance followed by Dunn’s multiple comparison test). (**c,d**) Immunohistochemical stainings for ADAM8 in human atherosclerotic lesions. (**c**) Representative images of a shoulder region (scale bar, 400 μm), a region rich in foamy macrophages (scale bar, 50 μm) and a negative control (**d**, scale bar, 200 μm) are shown.

Reanalysis of a previous microarray study^20^ (GSE7074) from our laboratory comparing human atherosclerotic plaque macrophages and resident macrophages derived from liver, lung and spleen revealed ADAM8 as one of the highest upregulated genes in plaque macrophages (Fig. 1a), which prompted us to investigate ADAM8 gene expression in different stages of human atherosclerotic plaque development. Tissue lysates from early, stable and unstable human lesions showed significantly increased ADAM8 mRNA expression in unstable lesions (Fig. 1b).

Immunohistochemical staining of ADAM8 in human carotid atherosclerotic plaques confirmed the expression of ADAM8 in lesions at the protein level. ADAM8 expression was not only found in leukocytes (as previously reported^13^), but also in luminal and microvascular endothelial cells and, potentially, vascular smooth muscle cells (suppl. Figure 1a and b). ADAM8 localized intensely to the shoulder regions of the plaque (Fig. 1c), an area mainly composed of inflammatory cells^21^.

### *Adam8* is mainly expressed in neutrophils and macrophages and modulates secretion of inflammatory mediators

**Figure 2:**
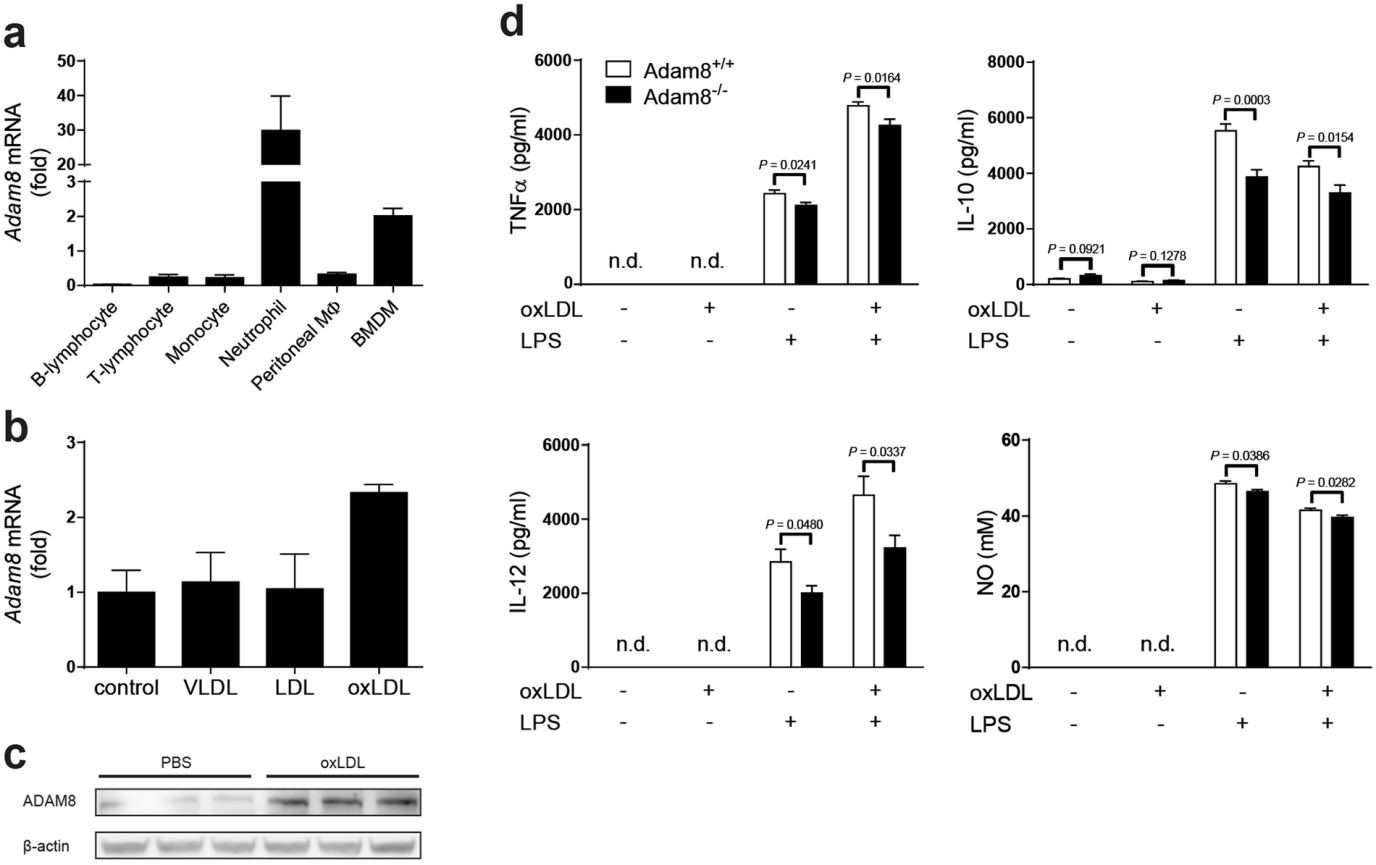
*Adam8* expression in leukocyte subsets and ADAM8 deficient BMDMs show reduced inflammatory response. (**a**) Relative *Adam8* mRNA expression in sorted murine C57Bl/6 blood B-lymphocytes, T-lymphocytes, monocytes, neutrophils, resident peritoneal macrophages and BMDMs (*n* = 4 mice). Fold changes of *Adam8*/*Gapdh* expression is shown. (**b**) *Adam8* expression in wildtype C57Bl/6 BMDMs exposed for 24 h to 0.25 μg/ml VLDL, LDL or oxLDL (*n* = 2 per group). (**c**) Western blot analysis of BMDMs stimulated with PBS or 0.25 μg/ml oxLDL (*n* = 3 per group). (**d**) Cytokine or nitric oxide (NO) secretion by *Adam8* ^+/+^ and *Adam8* ^−/−^ BMDMs that were pre-treated with PBS or 0.25 μg/ml oxLDL for 24 hours, followed by a 6 hours (TNFα and IL-10) or 24 hours (IL-12 and NO) incubation with PBS or 10 ng/ml LPS (*n* = 3 mice per group, nonparametric Mann-Whitney *U* test).

Leukocytes play a pivotal role in both human and murine atherosclerosis development^22^. In homeostatic conditions, expression of ADAM8 is mainly restricted to cells of the immune system^8^. We therefore sought to examine its expression in various leukocyte subsets isolated from wildtype C57Bl/6 mice. Interestingly, *Adam8* mRNA is mainly expressed in circulating neutrophils and, to a lower extent, in bone marrow-derived macrophages (BMDMs; Fig. 2a). In contrast, expression of *Adam8* mRNA in monocytes and B- and T-lymphocytes was barely detectable. ADAM8 expression was reported to increase under pathological conditions^11, 16–18^. Since macrophages are the main inflammatory cell type in mouse atherosclerotic plaques where they will be exposed to (modified) lipoproteins^23^, we investigated *Adam8* expression in BMDMs exposed to different types of lipoproteins. Interestingly, while *Adam8* mRNA levels were not affected by very low-density lipoproteins (VLDLs) or low-density lipoproteins (LDL), oxidized LDL increased ADAM8 mRNA (Fig. 2b) and protein (Fig. 2c) expression in BMDMs. This is in line with the pronounced ADAM8 expression in foamy macrophages of human atherosclerotic plaques (Fig. 1c). Since ADAMs play a crucial role in modulating inflammatory responses, we examined the role of ADAM8 in LPS-induced cytokine production by BMDMs. Interestingly, ADAM8 deficiency resulted in significantly reduced TNFα, interleukin (IL)-10 and IL-12 as well as nitric oxide (NO) secretion, both when BMDMs were pre-exposed to oxLDL followed by LPS or LPS alone (Fig. 2d).

### Hematopoietic ADAM8 deficiency does not affect size or composition of advanced atherosclerotic lesions

**Figure 3:**
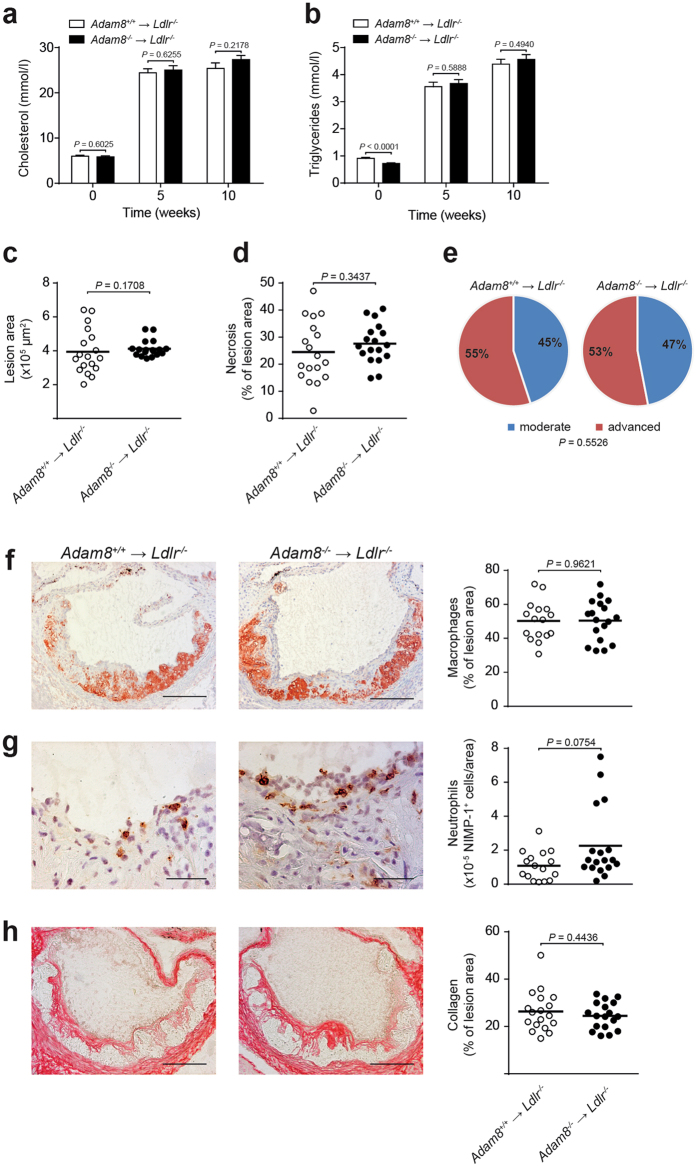
Hematopoietic ADAM8 deficiency in *Ldlr* ^−/−^ mice does not affect size or morphology of advanced atherosclerotic lesions. (**a,b**) Plasma cholesterol (**a**) and triglyceride (**b**) levels after 0, 5 and 10 weeks of western type diet (WTD) feeding in female *Ldlr* ^−/−^ chimeras with (*Adam8* ^−/−^ → *Ldlr* ^−/−^) or without (*Adam8* ^*+/+*^ → *Ldlr* ^−/−^) hematopoietic ADAM8 deficiency (*n* = 20 mice per genotype, parametric Student’s *t*-test). (**c, d**) Quantification of the aortic root lesion area (**c**, non-parametric Mann-Whitney *U* test) and necrotic core area (**d**, parametric Student’s *t*-test) of *Adam8* ^*+/+*^ → *Ldlr* ^−/−^ and *Adam8* ^−/−^ → *Ldlr* ^−/−^ mice (*n* = 18 mice per genotype) after 10 weeks of WTD. (**e**) Plaque progression stage (*n* = 51 aortic root atherosclerotic lesions per genotype) was scored (Fisher’s exact test). (**f**–**h**) Representative examples of (immuno)histochemical stainings and quantifications for MOMA-2^+^ macrophages (**f**, *n* = 18/16 mice, parametric Student’s *t*-test, scale bar, 200 μm), NIMP^+^ neutrophils (**g**, *n* = 18/16 mice, non-parametric Mann-Whitney *U* test, scale bar, 50 μm) and Sirius Red stained collagen (**h**, *n* = 18/16 mice, parametric Student’s *t*-test, scale bar, 200 μm) in the aortic root after 10 weeks of WTD feeding.

Considering its expression in hematopoietic cells and its role in modulating macrophage inflammatory responses, we investigated the effect of hematopoietic deficiency of ADAM8 on lesion development by reconstituting lethally irradiated female low-density lipoprotein deficient (*Ldlr* ^−/−^) mice with bone marrow from either ADAM8 deficient (*Adam8* ^−/−^ → *Ldlr* ^−/−^) mice or wildtype (*Adam8* ^+/+^ → *Ldlr* ^−/−^) littermate controls. The efficiency of the bone marrow transplantation (BMT) was 96% ± 0.1. After reconstitution, mice were fed a western type diet (WTD) for 10 weeks after which lesions development was assessed. No differences between genotypes were found in body weight (suppl. Figure 2a) or plasma cholesterol before and after WTD (Fig. 3a). Although at baseline triglyceride levels in *Adam8* ^−/−^ → *Ldlr* ^−/−^ mice were slightly decreased compared to *Adam8* ^+/+^ → *Ldlr* ^−/−^ mice, no differences were observed upon WTD (Fig. 3b). Furthermore, after recovery from the BMT, circulating leukocyte counts were analyzed by flow cytometry. Both at baseline (before WTD) and after 10 weeks of WTD the total number of CD45^+^ leukocytes in blood was significantly reduced in hematopoietic ADAM8 deficient mice compared to wildtype controls (suppl. Figure 2b and c). ADAM8 deficiency associated leukopenia affected almost all major leukocyte (sub)populations.

After 10 weeks of WTD feeding, atherosclerotic lesion area was not affected by hematopoietic ADAM8 deficiency, both in the aortic root (Fig. 3c) and brachiocephalic artery (suppl. Figure 2d). Furthermore, there was no change in necrotic core content between both groups in the aortic root (Fig. 3d). Routine pathological examination for atherosclerotic plaque progression (Fig. 3e) as well as (immuno)histochemical stainings for macrophage (Fig. 3e), neutrophil (Fig. 3f) and collagen content (Fig. 3g) showed no significant differences between *Adam8* ^−/−^ → *Ldlr* ^−/−^ and *Adam8* ^+/+^ → *Ldlr* ^−/−^ mice. Moreover, mRNA levels of *Arg1* and *Nos2*, were unchanged between both genotypes (suppl. Figure 2e), suggesting no difference in macrophage polarization. Moreover, we did not observe a compensatory upregulation of the related metalloprotease *Adam17*. Altogether, these data show that, despite reduced amounts of circulating leukocytes, hematopoietic ADAM8 deficiency does not influence advanced atherosclerotic lesion development or composition.

### Advanced atherosclerotic lesions are not affected by whole-body ADAM8 deficiency

Most ADAM family members are expressed as transmembrane proteases. ADAM8, however, also exists in a soluble form comprising the, functional, metalloproteinase domain only^24, 25^. Although significantly lower compared to wildtype controls, hematopoietic ADAM8 deficient mice still have considerable plasma levels of soluble ADAM8 (suppl. Figure 2f) most likely originating from non-hematopoietic cells which may have compensated for the hematopoietic deficiency.

**Figure 4:**
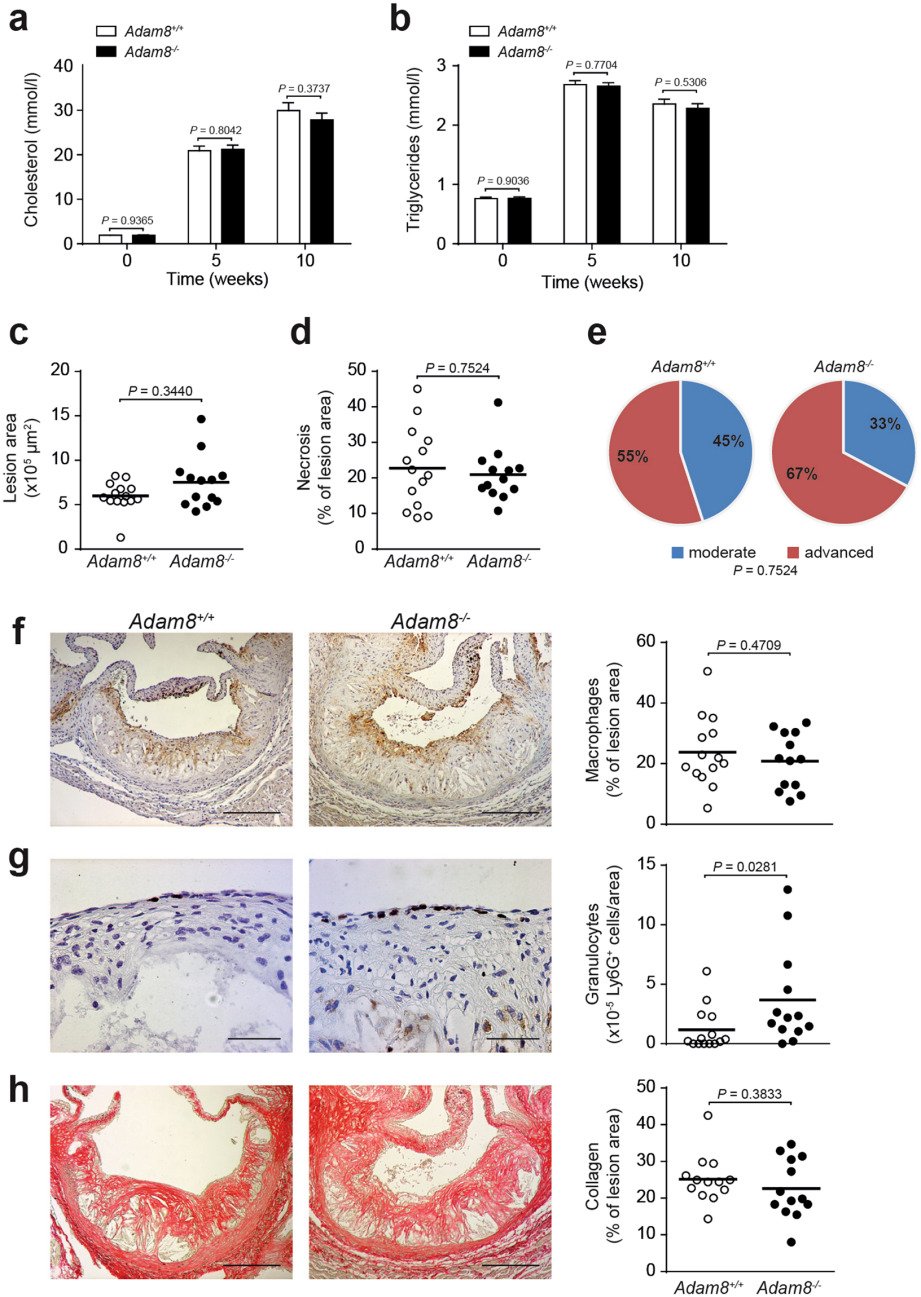
Whole-body ADAM8 deficiency has no effect on advanced plaque size or morphology. (**a**,**b**) Plasma cholesterol (**a**) and triglyceride (**b**) levels of female whole-body wildtype (*Adam8* ^+/+^) and *Adam8* ^−/−^ mice rendered hyperlipidemic by AAV8-PCSK9 gene transfer and subsequent western type diet (WTD) feeding, at start of WTD diet (0) and after 5 and 10 weeks (*n* = 16/14 mice, parametric Student’s *t*-test). (**c**,**d**) Quantification of the aortic root lesion area (**c**, *n* = 14/13 mice, nonparametric Mann-Whitney *U* test) and necrotic core area (**d**, *n* = 14/13 mice, nonparametric Mann-Whitney *U* test) of whole body *Adam8* ^+/+^ and *Adam8* ^−/−^ mice after 10 weeks of WTD feeding. (**e**) Plaque stage classification (*n* = 42/39 atherosclerotic lesions in the aortic root) was scored (Fisher’s exact test). (**f**–**h**) Representative examples of (immuno)histochemical stainings for MAC-3^+^ macrophages (**f**, *n* = 14/13 mice, parametric Student’s *t*-test scale bar, 200 μm), Ly6G^+^ granulocytes (**g**, *n* = 14/13 mice, nonparametric Mann-Whitney *U* test scale bar, 50 μm) and Sirius Red stained collagen (**h**, *n* = 14/13 mice, nonparametric Mann-Whitney *U* test scale bar, 200 μm) with quantification.

To exclude this possibility, we rendered female whole-body *Adam8* ^−/−^ and wildtype littermate controls prone to atherosclerosis by using a recently developed proprotein convertase subtilisin/kexin type 9 (PCSK9) overexpressing adeno-associated viral vector in combination with WTD feeding^26^. Indeed, PCSK9 overexpression raised both plasma cholesterol and triglyceride levels upon WTD feeding to a similar extent as in *Ldlr* ^−/−^ mice, but both were not found different between *Adam8* ^−/−^ and wildtype mice (Fig. 4a and b), despite a lower body weight in *Adam8* ^−/−^ mice after 10 weeks of WTD (suppl. Figure 3a). Additionally, blood leukocyte counts were measured using flow cytometry before animals were subjected to WTD feeding and at sacrifice (suppl. Figure 3b and c). In contrast to hematopoietic ADAM8 deficient mice, no leukopenia was observed and whole-body ADAM8 deficiency even resulted in slightly increased T cell and NK cell counts under hyperlipidemic conditions.

Surprisingly, even whole-body ADAM8 deficiency did not affect atherosclerotic lesion size in the brachiocephalic artery (suppl. Figure 3d) and aortic root (Fig. 4c). Similar to hematopoietic ADAM8 deficient mice, necrotic core content (Fig. 4d), plaque stage (Fig. 4e), as well as (immuno)histochemical staining of macrophage (Fig. 4f) and collagen content (Fig. 4h) showed no difference in whole body *Adam8* knockout mice compared to wildtype controls. Moreover, *Arg1, Nos2* and *Adam17* mRNA expression was similar between both genotypes (suppl. Figure 3e). However, the amount of granulocytes was significantly increased in ADAM8 deficient mice compared to wildtype mice (Fig. 4g). Collectively, these data show that both hematopoietic as well as whole-body ADAM8 deficiency does not affect advanced atherogenesis in mice.

## Discussion

This is the first study that investigated the role of ADAM8 in atherosclerosis development. Although, we clearly showed that ADAM8 expression is increased in unstable human atherosclerotic lesion, we did not observe any effects of hematopoietic nor whole-body ADAM8 on advanced atherosclerosis development in mice.

Originally identified in a human macrophage cell line^27^, ADAM8 is mainly expressed in most cells of hematopoietic origin, both under physiological as well as inflammatory conditions^13, 28^. Here, we show that in mice, under physiological conditions, *Adam8* indeed is highly expressed in circulating neutrophils and to a lower extent in bone marrow derived macrophages. In contrast, *Adam8* is hardly expressed by monocytes and lymphocytes, in agreement with open access mouse leukocyte expression databases (immgen.org). Similarly, in humans ADAM8 was also seen to be expressed by neutrophils^25^ and monocytes^13, 29^, while T lymphocytes lack ADAM8 expression^13^. Interestingly, however, human B lymphocytes do express ADAM8, albeit to a lower extent compared to human monocytes^13^. These findings suggest species differences in hematopoietic ADAM8 expression, at least in T and B lymphocytes. Next to neutrophils, macrophages express higher levels of *Adam8* than monocytes and lymphocytes under physiological conditions. Interestingly, peritoneal macrophages, which are a subset of resident macrophages, express similar low *Adam8* levels as monocytes. The difference in *Adam8* expression between freshly differentiated macrophages from bone marrow cells and resident macrophages might be due to the difference in their local microenvironment which initiate different transcriptional programs^30^ or their mode of differentiation^31^.

The atherosclerotic plaque harbors multiple environmental cues which dynamically regulate the expression profiles of macrophages^32^. Previously, it has been shown that ADAM8 gene and protein expression increase upon atherosclerotic lesion development in humans, and that its expression co-localized with plaque smooth muscle cells and macrophages^33^. In this study, we show that ADAM8 is especially abundant in the plaque shoulder regions, which are rich in leukocytes, especially (foamy) macrophages. Furthermore, oxLDL, which is abundantly present in atherosclerotic lesions and can modify macrophage phenotype and function^23^, was seen to upregulate ADAM8 expression, unlike LDL and VLDL. Interestingly, ADAM8 deficient mouse macrophages have an attenuated secretion of inflammatory mediators, including TNF, a proposed substrate of ADAM8^34^. Despite ADAM17 being the main TNF sheddase both *in vitro* and *in vivo* ^35, 36^, ADAM8 deficiency has a significant contribution to TNF release *in vitro*. We found both pro-and anti-inflammatory cytokines to be reduced, i.e. no clear M1 or M2 profile, in line with unchanged *Nos2* and *Arg-1* expression in plaques *in vivo*.

Remarkably, despite the absence of any effects of hematopoietic ADAM8 deficiency on atherosclerosis development, these bone marrow transplanted mice displayed a slight decrease in triglyceride levels at baseline and mild leukopenia both under normo- and hyperlipidemic conditions. Although only evident during recovery after bone marrow transplantation, the potential role of hematopoietic ADAM8 deficiency in triglyceride metabolism remains to be determined. Regarding the observed leukopenia, ADAM8 has previously been shown to regulate the surface expression of the adhesion molecules L-selectin and PSGL-1 through shedding^25, 28^. Interestingly, ligation of PSGL-1 on hematopoietic progenitor cells to P-selectin results in suppression of hematopoiesis^37^. Additionally, L-selectin may function as a retention cue for granulocytes to remain in the bone marrow^38, 39^. Although ADAM8 might indirectly affect both processes, via these two substrates, the fact that whole-body ADAM8 deficient mice failed to display leukopenia does not plead for this notion. Another possibility for this discrepancy might lie in a role for ADAM8 in the homing of progenitor cells towards the bone marrow niche after BMT.

Furthermore, ADAM8-mediated shedding of the adhesion molecules L-selectin and PSGL-1 may also control neutrophil recruitment^25, 28^. ADAM8 deficiency results in increased surface expression of both molecules, which will lead to increased tethering of neutrophils to endothelial cells^40^. In line, atherosclerotic lesions of whole-body ADAM8 deficient mice contained increased amounts of granulocytes compared to their respective controls. In contrast, hematopoietic ADAM8 deficiency did not result in an accumulation of neutrophils in atherosclerotic lesions, albeit this might be due to the presence of sufficient levels of plasma sADAM8, which also has the capacity to cleave transmembrane proteins, like L-selectin. Indeed, transgenic mice that overexpressed the soluble form of ADAM8 display a reduction in surface expression of L-selectin and concomitantly a decrease in leukocyte accumulation in the peritoneal cavity after receiving an inflammatory stimulus^41^. Similarly, overexpression of ADAM8 might also attenuate leukocyte recruitment towards atherosclerotic lesions, and thus reduce atherosclerotic burden. On the other hand, however, overexpression of ADAM8 may also increase the inflammatory response of macrophages and other cell types that have the capacity to secrete TNFα^34^. Therefore, ADAM8 affects processes that inhibit or exacerbate atherosclerosis development, which could explain why we did not observe any change in plaque size or morphology in female ADAM8 deficient mice. Note, although no reports on sex specific effects of ADAM8 have been reported, we cannot exclude the possibility that ADAM8 differentially affects atherosclerosis development in males.

In addition to the potential dual effects of ADAM8 in atherosclerosis, other ADAMs with a similar substrate profile as ADAM8, such as ADAM17^42^, might at least partly compensate for the loss of ADAM8. ADAM17 expression was unchanged between ADAM8 wildtype and knockout mice in both atherosclerosis models, suggesting there is no overcompensation for the loss of ADAM8 deficiency although this does not rule out any functional compensation.

Furthermore, neutrophils are involved in the process of atherosclerosis, primarily during the initiation of lesion development^43^. Therefore, it cannot be excluded that ADAM8 might have an effect at earlier developmental stages, since all atherosclerotic lesions in this study were classified as moderate to advanced.

We and others^33, 44^ have shown that ADAM8 expression is increased in advanced human atherosclerotic lesions. Next to its expression in macrophages, especially in foam cells located at the shoulder region of the atherosclerotic lesion, ADAM8 was also expressed in plaque stromal cells, including endothelial cells and, potentially, smooth muscle cells, though the role of ADAM8 in these vascular cell types is less defined. Risk allele carriers have increased serum levels of soluble ADAM8 and an increased risk of myocardial infarction^19^, which shows that ADAM8 may still have clinical potential.

In conclusion, while ADAM8 affects inflammatory responses *in vitro*, our data argue against a critical role for both hematopoietic and whole-body ADAM8 in atherosclerosis development in female mice, at least in advanced stages of the disease. However, ADAM8 may still be useful as a diagnostic/prognostic biomarker to distinguish between stable and unstable atherosclerotic lesions in humans, though further research is needed.

## Materials and Methods

### Microarray and quantitative PCR analysis of human tissues

Human carotid artery plaque tissue^45^ and nonatherosclerotic (lung, liver, spleen) tissues^20^ were obtained by endarterectomy or autopsies, respectively, as previously described. Collection, storage and use of tissue in the Maastricht Pathology Tissue Collection (MPTC) and patient data confidentiality were performed after informed consent and in agreement with the ‘Code for Proper Secondary Use of Human Tissue in the Netherlands’ and in accordance with the guidelines of, and approved by the medical and ethical committee of Maastricht University Medical Centre, Maastricht, The Netherlands. Sample processing, macrophage isolation and microarray hybridization and analysis of the differences between carotid plaque, lung, liver and spleen macrophages was performed as previously described (Gene Expression Omnibus database accession number GSE7074)^20^. Carotid lesion segments for quantitative PCR analysis were snap-frozen and subsequently RNA was isolated using the guanidine isothiocyanate/CsCl method as previously described^45^. RNA was further purified and concentrated using RNeasy mini columns (Qiagen). Total RNA was normalized and reverse transcribed using iScript (Biorad). Quantitative PCR (qPCR) was performed using 10 ng cDNA, 300 nM of each primer, and SensiMix (Quantace-Bioline). All gene expression levels were corrected for cyclophilin A and ß-actin as housekeeping genes.

### Immunohistochemistry of human tissues

Human carotid endarterectomy segments were fixed in paraformaldehyde and paraffin embedded. Sections were incubated with a primary antibody against human ADAM8 (AF1031, R&D systems), followed by detection with a biotin-labelled rabbit anti-goat antibody (E0466, Dako) and Vector Red ABC kit (Vector Labs, CA, USA). Atherosclerotic lesions were classified as introduced by Virmani *et al*.^46^. Pathological intimal thickening or xanthomata were defined as ‘early’, thick fibrous cap atheroma ‘stable’ and lesions with a thrombus or intraplaque hemorrhage ‘unstable’ lesions.

### Animals

Mouse experiments were approved by the Animal Ethics Committee of Maastricht University, the Netherlands (permit number 2012-065), and were performed in compliance with the Dutch government guidelines. Female *Ldlr* ^−/−^ mice were obtained from an in-house breeding colony, originally derived from Charles River (Wilmington, MA, USA). Female *Adam8* knockout (*Adam8* ^−/−^) and wildtype (*Adam8* ^+/+^) littermate control mice on a C57Bl/6 background were previously described and generously provided by Dr. J. Bartsch^15^.

### Peritoneal macrophage isolation

Resident peritoneal macrophages were obtained by flushing the peritoneal cavity with ice-cold phosphate buffered saline (PBS) followed by culturing in RPMI 1640 Glutamax containing 10% (vol/vol) heat inactivated fetal calf serum (FCS), penicillin (100 U/ml), streptomycin (100ug/ml), and L-glutamine 2 mM (all GIBCO Invitrogen, Breda, the Netherlands). After overnight attachment, cells were washed three times with RPMI 1640 Glutamax to remove non-adherent cells. Adherent cells were dissolved in TRIzol reagent (Life Technologies) and stored at −20 °C until further use.

### Bone marrow-derived macrophage isolation and culture

Bone marrow cells were isolated from femurs and tibiae of either *Adam8* ^−/−^ or wildtype littermate controls. Cells were cultured in RPMI 1640 Glutamax (GIBCO Invitrogen, Breda, the Netherlands) with 10% (vol/vol) heat-inactivated FCS (Bodinco B.V. Alkmaar, the Netherlands), penicillin (100 U/ml) and streptomycin (100 μg/ml) supplemented with 15% L929-conditioned medium (LCM) for 8 days to generate bone-marrow derived macrophages (BMDMs). BMDMs were seeded at 0.35 × 10^6^ cells per well in 24 wells plates and incubated 6-24 hours with 10 ng/ml LPS (E. Coli 055:B5, Sigma). Alternatively, BMDMs were incubated for 24 hours with 25 μg/ml very-low density lipoprotein (VLDL), low density lipoprotein (LDL) or oxidized LDL (oxLDL). VLDL and LDL were isolated from human serum^47^ and LDL oxidatively modified by CuSO4 as previously described^48^. Supernatants were collected and stored at −20 °C until further use.

### Fluorescence-activated cell sorting

Leukocyte subsets were isolated from blood collected from wildtype C57Bl/6 mice using a FACS Aria (BD Biosciences). Erythrocytes were removed by incubation with erylysis buffer (155 mM NH_4_Cl and 10 mM KHCO_3_). Leukocytes were defined as CD45^+^ (Biolegend), T-lymphocytes as CD45^+^ CD3^+^ (eBioscience) NK1.1^−^ (BD), B-lymphocytes as CD45^+^ CD3^−^ NK1.1^−^ B220^+^ (BD), granulocytes as CD45^+^ CD3^−^ NK1.1^−^ B220^−^ CD11b^+^ (BD) Ly6G^+^ (BD) and monocytes as CD45^+^ CD3^−^ NK1.1^−^ B220^−^ CD11b^+^ Ly6G^−^.

### Quantitative PCR in murine cells

RNA from FACS sorted cells and murine aortic arches was isolated using TRIzol (Life Technologies) or RNeasy mini kit (Qiagen), respectively, according to the manufacturer’s instructions. RNA (500 ng) was reverse transcribed using the iScript cDNA Synthesis Kit (Biorad). Quantitative PCR was performed using 10 ng cDNA, 300 nmol/L of each primer and Sensimix SYBR Green (Biorad) in a total volume of 12 μL. All gene expression levels were expressed relative to *Gapdh* as housekeeping genes. Primer sequences are available upon request.

### ELISA

TNF, IL-10 (after 6 h LPS) and IL-12p40 (after 24 h LPS) levels in BMDM-derived conditioned medium were measured with ELISA kits (Invitrogen) according to the manufacturer’s instructions. Plasma soluble ADAM8 was measured using a commercial ELISA kit (Hoelzel Diagnostica, Cologne, Germany) according the manufacturer’s instructions. Analysis was performed using a micro-plate reader (Biorad).

### NO assay

NO_2_^−^ concentrations were determined in LPS-induced BMDM-derived conditioned medium using Griess reagent (2.5% H_3_PO_4_, 1% sulphanilamide, 0.1% naphthalene diamine dihydochloride) and measured at 540 nm (benchmark microplate reader, Biorad).

### Western Blot analysis

After a 24 hours incubation with PBS or 25 μg/ml oxLDL, BMDMs were treated lysis buffer (150 mM NaCl, 50 mM Tris, 10 mM EDTA, 1% NP-40, 8.7% glycerol, 0.1% SDS) supplemented with 1x Complete Inhibitor (Roche). After centrifugation at 16.000 x *g* for 5 min, supernatants were collected and protein concentration was determined using bicinchoninic acid (BCA) assay (Thermofisher). Supernatants were investigated by reducing SDS-PAGE as previously described^4^. Proteins were transferred onto nitrocellulose membrane (GE Healthcare Life Sciences). Membranes were blocked with 5% (w/v) non-fat dry milk in Tris buffered saline with 0.1% Tween (TBS-T) for 1 hour, cut into two horizontal strips based on the predicted molecular weights of the target proteins, and then probed with primary antibody against murine ADAM8^49^ (Abcam) or β-actin (Abcam) overnight, followed by appropriate horseradish peroxidase conjugated secondary antibodies. Chemiluminescence was detected using ImageQuant LAS 4000 mini (GE Healthcare Life Sciences). Images of full-length blots are shown in suppl. Figure 4.

### Bone marrow transplantation and atherosclerotic lesion analyses

Female wildtype and *Adam8* ^−/−^ mice (aged 10 to 12 weeks) were used as donor mice (*n = *4 per group). Female *Ldlr* ^−/−^ recipient mice (10 weeks old, *n* = 18 per group), backcrossed onto a C57Bl/6 background for >10 generations, were obtained from in-house breeding. Bone marrow transplantations (BMT) were performed as described elsewhere^45^. Briefly, *Ldlr* ^−/−^ mice were lethally irradiated with 6 Gy a day before and on the day of transplantation. Mice were transplanted with 5 × 10^6^ bone marrow cells isolated from wildtype or *Adam8* ^−/−^ mice. After a recovery period of 5 weeks after transplantation, mice were given a western type diet (WTD) containing 0.25% cholesterol (Special Diets Services, Witham, Essex, UK). Before WTD feeding, mice were fasted for 4 hours, after which blood samples were drawn from the tail vein for analyses of plasma lipids, chimerism, soluble ADAM8 and leukocytes. Additional blood analyses were performed at 5 and 10 weeks of WTD, after 4 hours fasting. After 10 weeks of WTD feeding, mice were anesthetized, euthanized and perfused with PBS containing nitroprusside (0.1 mg/ml, Sigma).

Mouse hearts were dissected and snap-frozen in optimum cutting temperature medium. Serial cryosections (7 μm) of the aortic root and brachiocephalic artery were fixed in acetone and stained with toluidine blue for morphometric analysis of plaque and necrotic core area (defined as acellular regions). Total plaque area per mouse was defined as the average plaque area of four consecutive toluidine blue-stained sections at 42 μm intervals. Plaque stages were determined as previously described^50^, with slight modifications. Plaques were classified as early (foam cell rich, but lacking a necrotic core), moderately advanced (containing a fibrotic cap and often a necrotic core, but no medial macrophage infiltration) and advanced lesions, typified by medial macrophage infiltrates, elastic lamina degradation and more pronounced necrosis and fibrosis. MOMA-2 (an antibody recognizing monocytes/macrophages; gift from G. Kraal), NIMP-1 (neutrophil specific antibody directed against Ly6G; gift from P. Heeringa) and Sirius Red (Sigma) staining was used for the detection of monocytes/macrophages, neutrophils and collagen, respectively. Pictures were taken using a Leica DM3000 light microscope (Leica Microsystems, Wetzlar, Germany) and sections were analyzed in a blinded manner using Adobe Photoshop CS6 software (Adobe, San Jose, US).

### Atherosclerotic lesion analysis in whole-body ADAM8 deficient mice

Female wildtype and *Adam8* ^−/−^ mice (aged 10 to 12 weeks; *n = *16 and 14, respectively) were rendered prone to atherosclerosis by a single intravenous injection of adeno-associated virus serotype 8 containing D377Y-murine PCSK9 (AAV-PCSK9; 1 × 10^11^ vector genomes per mouse; as described previously^26^), followed by WTD feeding (0.25% cholesterol; Special Diets Services, Witham, Essex, UK). Blood was collected from the tail vein for analyses of plasma lipids at baseline (before WTD) and after 5 and 10 weeks of WTD feeding after 4 hours fasting. After 10 weeks of WTD feeding, mice were anesthetized, euthanized and perfused with PBS containing nitroprusside (0.1 mg/ml, Sigma). Mouse hearts were excised and fixed overnight in 1% paraformaldehyde. Serial paraffin sections of the aortic root and brachiocephalic artery were cut (4 μm) and stained with hematoxylin and eosin (H&E, Sigma) for morphometric analysis of plaque and necrotic core area (defined as acellular regions), and plaque staging, as described in the BMT section. Total plaque area per mouse was defined as the average plaque area of five consecutive H&E-stained sections at 20 μm intervals. Atherosclerotic lesions were further characterized for macrophage (MAC3, BD), granulocyte (Ly6G, Becton & Dickenson) and collagen (Sirius Red) content. Similar as to the BMT setup, pictures were taken using a Leica DM3000 light microscope and sections were analyzed in a blinded manner using Adobe Photoshop CS6 software.

### Flow cytometry analyses and blood lipid analyses

Absolute circulating leukocyte subset numbers were determined by flow cytometry calibrated using Trucount Beads (BD, New Jersey, US). Blood was collected at the start (t = 0) and after 10 weeks of WTD. Erythrocytes were removed by incubation with erylysis buffer (155 mM NH_4_Cl and 10 mM KHCO_3_). Leukocytes were defined as CD45^+^ (Biolegend), T-lymphocytes as CD45^+^ CD3^+^ (eBioscience) NK1.1^−^ (BD), NK cells as CD45^+^ CD3^−^ NK1.1^+^, B-lymphocytes as CD45^+^ CD3^−^ NK1.1^−^ B220^+^ (BD) granulocytes as CD45^+^ CD3^−^ NK1.1^−^ B220^−^ CD11b^+^ (BD) Ly6G^+^ (BD) and monocytes as CD45^+^ CD3^−^ NK1.1^−^ B220^−^ CD11b^+^ Ly6G^−^. Monocyte subsets were further distinguished based on the degree of Ly6C expression. Data were acquired using a FACS Canto II (BD Bioscience) and analyzed with FACSdiva software (BD Bioscience).

Blood was collected at the start (t = 0) and after 5 and 10 weeks of WTD. Plasma was separated by centrifugation (2100 x *g*, 10 minutes, 4 °C), and stored at -80 °C until further use. Plasma cholesterol and triglycerides were determined using standard enzymatic kits (Cholesterol FS’10; Triglycerides FS 5’ Ecoline; Diagnostic Systems GmbH, Holzheim, Germany) according to the manufacturer’s instructions.

### Statistics

Data are presented as mean ± SEM. All statistical analyses were performed using the Prism software (GraphPad Software version 5, San Diego, CA, USA). Differences between two groups or more were evaluated for statistical significance with the appropriate Student’s *t* test, Mann-Whitney *U* test, Fisher’s exact test or one-way analysis of variance followed by Dunn’s multiple comparisons test, respectively. *P* values of less than 0.05 were considered significant.

## Electronic supplementary material

Supplemental figures 1, 2, 3 and 4
