# Supplementary material for: Whole body and hematopoietic ADAM8 deficiency does not influence advanced atherosclerotic lesion development, despite its association with human plaque progression
Source: Sci Rep. 2017 Sep 15;7:11670. doi: 10.1038/s41598-017-10549-x (PMC5601942; doi:10.1038/s41598-017-10549-x)
Supplement: Supplementary file 1 — Supplemental figures 1, 2, 3 and 4 [file 41598_2017_10549_MOESM1_ESM.pdf]

Supplementary information

Whole body and hematopoietic ADAM8 deficiency does not influence advanced atherosclerosis development, despite its association with human plaque progression

Kosta Theodorou, Emiel P.C. van der Vorst, Marion J. Gijbels, Ine M.J. Wolfs, Mike Jeurissen, Thomas L. Theelen, Judith Sluimer, Erwin Wijnands, Jack P. Cleutjens, Yvonne Jansen, Christian Weber, Andreas Ludwig, Jacob F. Bentzon, Jörg W. Bartsch, Erik A.L. Biessen, Marjo M.P.C. Donners

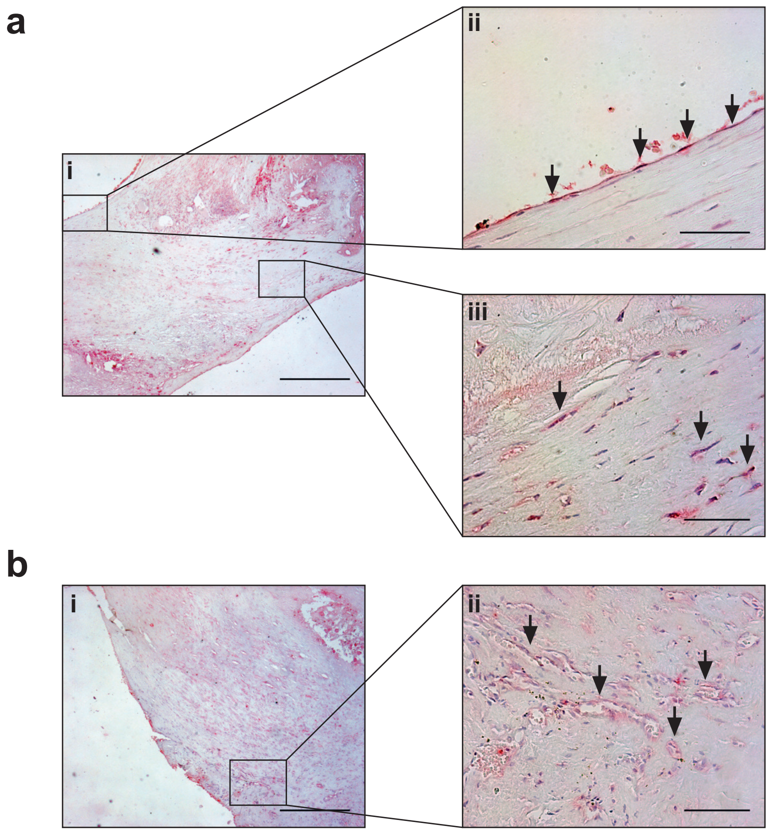

Supplemental figure 1. ADAM8 is expressed in human atherosclerotic lesions.
(**a-i**) Immunohistochemical staining of ADAM8 in a human atherosclerotic lesion (scale bar, 400 µm). Representative images of (**a-ii**) luminal endothelial cells (scale bar, 50 µm) and (**a-iii**) smooth muscle cells (scale bar, 50 µm; indicated by black arrows) are shown. (**b**) Representative immunohistochemical staining of ADAM8 in (**b-i**) human atherosclerotic lesion (scale bar, 400 µm) and (**b-ii**) microvessels is shown (scale bar, 100 µm).

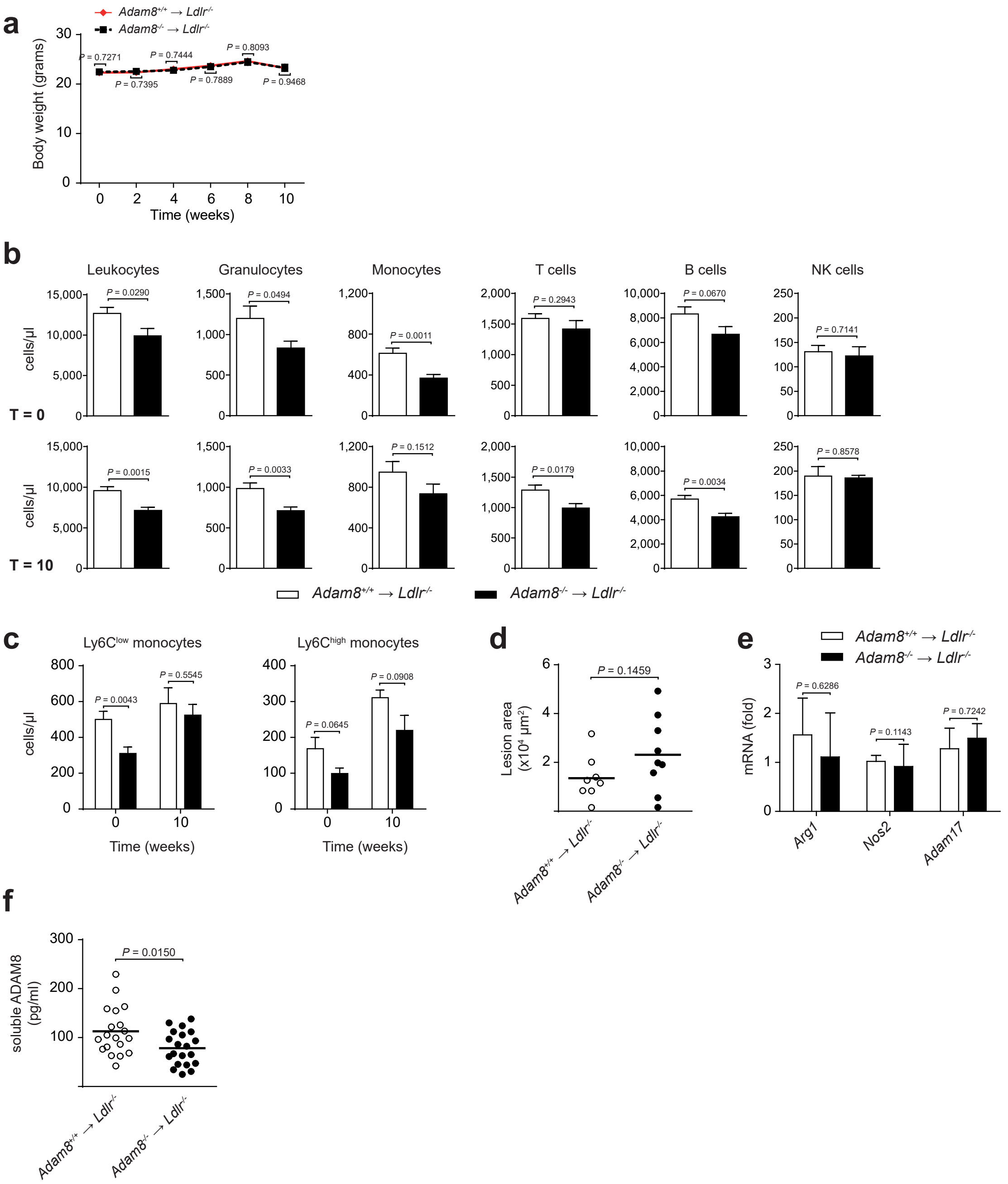

Supplemental figure 2. Hematopoietic ADAM8 deficiency reduces total blood leukocyte levels, but does not affect lesion size in the brachiocephalic artery.

(**a**) body weight over the course of 10 weeks of western type diet (WTD) in *Ldlr*$^{-/-}$ chimeras with (*Adam8*$^{-/-}$→*Ldlr*$^{-/-}$) or without (*Adam8*$^{+/+}$→*Ldlr*$^{-/-}$) hematopoietic ADAM8 deficiency (*n* = 20 mice per group, parametric Student's *t* test). (**b**) Blood counts of several leukocyte subsets at baseline (T = 0; *n* = 10 mice per group) and after 10 weeks of WTD feeding (T = 10; *n* = 10 mice per group, parametric Student's *t* test). (**c**) Ly6C^{low} and Ly6C^{high} monocyte subsets in blood at baseline and after 10 weeks of WTD (*n* = 10 mice per group, parametric Student's *t* test). (**d**) Quantification of the brachiocephalic artery lesion area in *Adam8*$^{-/-}$→*Ldlr*$^{-/-}$ and *Adam8*$^{+/+}$→*Ldlr*$^{-/-}$ mice (*n* = 8/9 mice) after 10 weeks of WTD (nonparametric Mann-Whitney *U* test). (**e**) mRNA expression levels in aortic arches of *Adam8*$^{-/-}$→*Ldlr*$^{-/-}$ and *Adam8*$^{+/+}$→*Ldlr*$^{-/-}$ (*n* = 5/8 mice) after 10 weeks of WTD (nonparametric Mann-Whitney *U* test). (**f**) plasma soluble ADAM8 protein 10 weeks of WTD feeding in *Adam8*$^{-/-}$→*Ldlr*$^{-/-}$ and *Adam8*$^{+/+}$→*Ldlr*$^{-/-}$ mice (*n* = 19/20 mice, nonparametric Mann-Whitney *U* test).

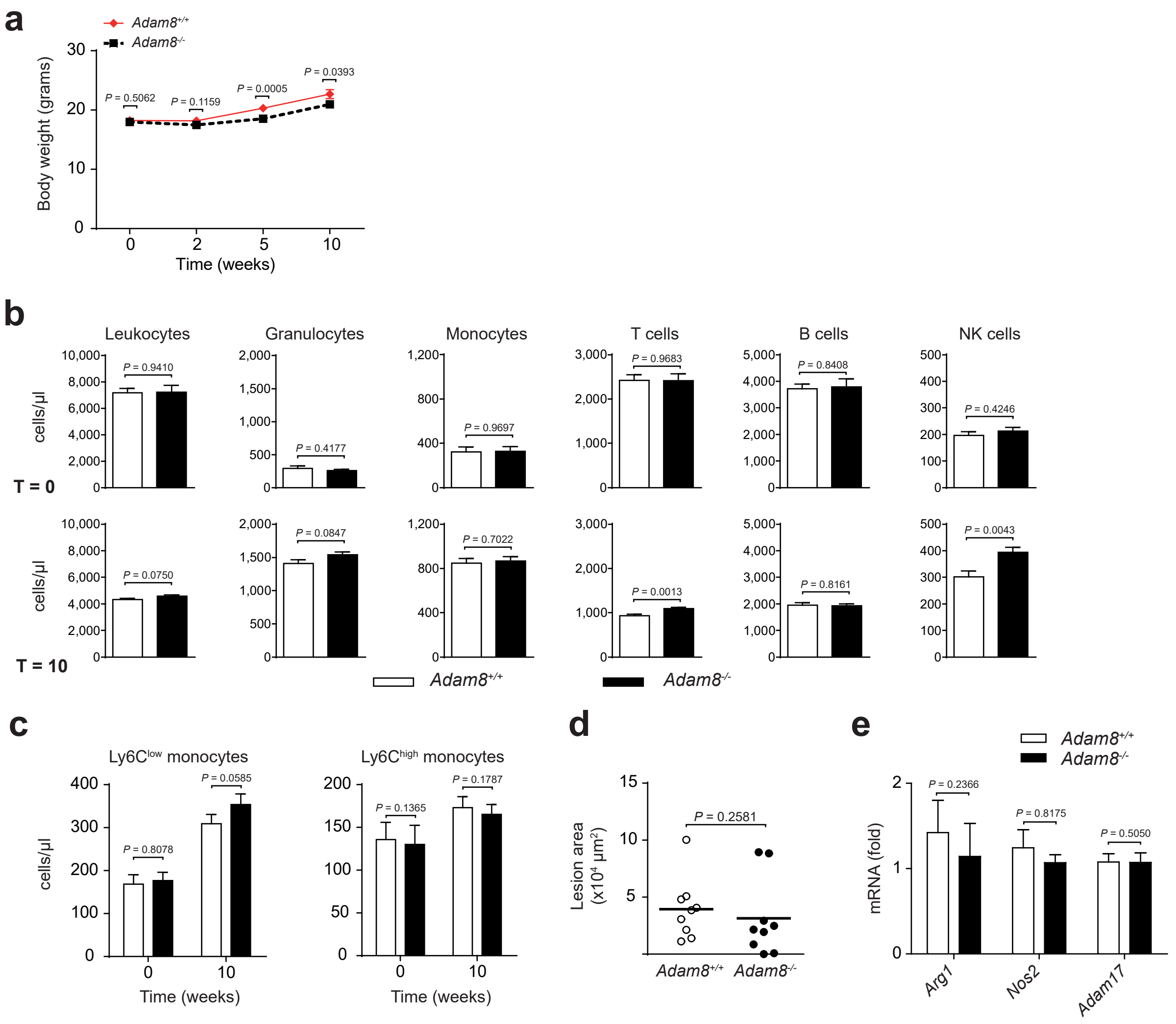

Supplemental figure 3. Whole-body ADAM8 deficient mice display increased T- and NK-cells after 10 weeks of WTD, but no change in brachiocephalic artery lesion size.

(**a**) body weight over the course of 10 weeks of western type diet (WTD) in ADAM8 deficient ($Adam8^{-/-}$) and wildtype ($Adam8^{+/+}$) mice (n = 16/14 mice, nonparametric Mann-Whitney *U* test). (**b**) Blood counts of several leukocyte subsets at baseline (T = 0; n = 10 mice per group) and after 10 weeks of WTD (T = 10; n = 10 mice per group, parametric Student’s *t* test). (**c**) Ly6C^{low} and Ly6C^{high} monocyte subsets in blood at baseline and after 10 weeks of WTD (n = 10 mice per group, parametric Student’s *t* test). (**d**) Quantification of the brachiocephalic artery root lesion area of $Adam8^{+/+}$ and $Adam8^{-/-}$ mice (n = 9 mice per group) after 10 weeks of WTD feeding (nonparametric Mann-Whitney *U* test). (**e**) mRNA expression levels in aortic arches of $Adam8^{+/+}$ and $Adam8^{-/-}$ (n = 14/13 mice) after 10 weeks of WTD (nonparametric Mann-Whitney *U* test).

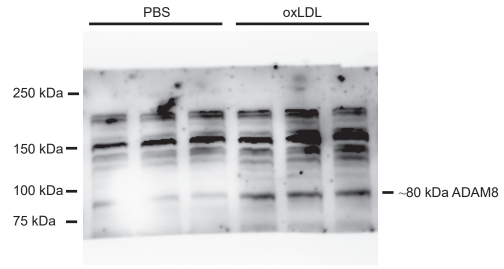

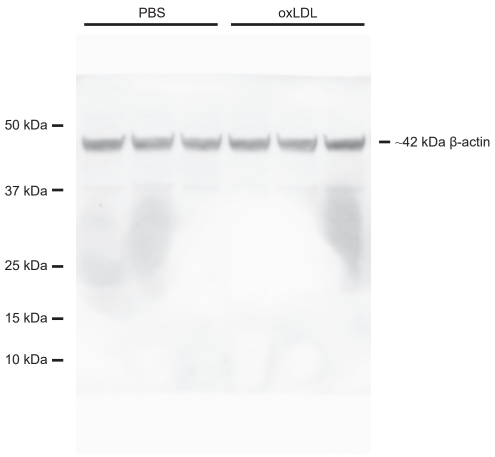

Supplemental figure 4. Images of full-length blots of figure 2c.
